# Large‐scale proteomics to enable precision medicine for Alzheimer’s disease

**DOI:** 10.1002/alz.090536

**Published:** 2025-01-09

**Authors:** Bart Smets, Asif Emon, Jon Greene, Pablo García‐González, Raquel Puerta, Alfredo Cabrera Socorro, Maarten Timmers, Agustin Ruiz, Christopher D Whelan

**Affiliations:** ^1^ Janssen Research & Development, A Division of Janssen Pharmaceutica, Neuroscience Therapeutic Area, Beerse Belgium; ^2^ Rancho Biosciences, Remote, CA USA; ^3^ Ace Alzheimer Center Barcelona – International University of Catalunya (UIC), Barcelona Spain; ^4^ Janssen Research & Development, LLC, a Johnson & Johnson company, Boston, MA USA

## Abstract

**Background:**

Precision neuroscience is emerging as a transformative approach that aims to identify the right treatment for the right patient at the right time. To enable this, it is important to move beyond the categorization of patients based on clinical symptoms towards a biological definition of disease. For Alzheimer’s disease, significant progress has been made in this direction, with the development of the “A/T/N” biomarker framework that classifies patients based on underlying pathophysiology.

**Method:**

It is essential to further characterize the molecular processes that drive disease progression within individual patients and to correctly stage those processes along the disease trajectory. Therefore, we are bringing together large biofluids proteomics datasets from different neurodegenerative disease cohorts. Analyses leverage Cox proportional‐hazards regressions to identify predictors of disease progression and, hypothesis‐driven (GSVA) and hypothesis‐free approaches (WGCNA, consensus clustering) to characterize pathological processes and disease subtypes.

**Result:**

Here, we present analyses of CSF proteomics data from the Spanish ACE Alzheimer’s disease cohort (n = 1321). MCI‐to‐dementia conversion analyses both replicate previous studies as well as identify novel biomarker candidates that might further augment the A/T/N framework (e.g. MMP10). We also provide an independent validation of work from Tijms et al. [1] that identified 3 different Alzheimer’s disease subtypes which are characterized by elevated levels of proteins associated with either (i) neuronal plasticity, (ii) innate immunity or (iii) blood‐brain barrier dysfunction. Extending upon these findings, we show that subtype 3 is characterized by an increase in CSF/plasma albumin ratio, further supporting the blood‐brain barrier dysfunction hypothesis. The subtypes stratify patients beyond the A/T/N categorization. The different subtypes might be valuable to increase precision.

**Conclusion:**

Large‐scale proteomics is a crucial tool to accelerate the development of novel precision treatments for Alzheimer’s disease, enabling the identification of novel fluid biomarkers for patient identification, stratification and disease progression, the characterization of molecularly defined disease subtypes, and the discovery of novel drug targets that are linked to specific patient populations.

[1] Tijms et al. Pathophysiological subtypes of Alzheimer’s disease based on cerebrospinal fluid proteomics. Brain 143, 3776–3792 (2020).

